# In Silico and In Vitro Evaluation of Quercetin Metabolites Binding to Inflammatory Target Proteins

**DOI:** 10.3390/ph19050655

**Published:** 2026-04-22

**Authors:** Rümeysa Yücer, Marie Ellen Periasamy, Axel Guthart, Angela Schröder, Gerhard Bringmann, Thomas Efferth, Joelle C. Boulos

**Affiliations:** 1 Department of Pharmaceutical Biology, Institute of Pharmaceutical and Biomedical Sciences, Johannes Gutenberg University, Staudinger Weg 5, 55128 Mainz, Germanyaguthart@students.uni-mainz.de (A.G.);; 2 Theophrastus Paracelsus Foundation, 64367 Mühltal, Germany; 3 Institute of Organic Chemistry, University of Wuerzburg, Am Hubland, 97074 Würzburg, Germany

**Keywords:** quercetin metabolites, anti-inflammation, NLRP3, microscale thermophoresis, molecular docking

## Abstract

**Background/Objectives**: The most abundant flavonoid, quercetin, which is mostly found as glycosides, is widely distributed in plants. Quercetin is rapidly metabolized, having a short half-life in the blood circulation, and forms its conjugates by undergoing ring cleavage of the benzopyranone ring system. Despite its fast clearance in the body, quercetin was demonstrated to have clinically anti-inflammatory, cardioprotective, antidiabetic, and anti-obesity activities. This study aimed to determine whether quercetin itself or its metabolites are responsible for these activities. **Methods**: We performed molecular docking of 27 metabolites, including quercetin itself, against ten inflammation-related proteins in silico. We then conducted microscale thermophoresis (MST) of selected metabolites towards the NLRP3 inflammasome. **Results**: Overall, Phase II metabolites yielded better binding energies compared to the metabolites formed by degradation. MST results revealed that isorhamnetin, the 4-*O*-methylated metabolite of quercetin, gave the best results, with a binding affinity (K_D_ value) of 16.12 ± 5.16 µM, even better than quercetin itself, which has a binding affinity of 44.84 ± 4.21 µM. Glucuronide metabolites of quercetin (isorhamnetin 3-*O*-glucuronide, quercetin 7-*O*-glucuronide, and quercetin 3-*O*-glucuronide) were found to bind to the inflammasome protein with low binding affinities, whereas small degradation products (hippuric acid and 3,4-dihydroxytoluene) did not bind at all. **Conclusions**: These results suggest that Phase II metabolites, specifically isorhamnetin, may contribute more significantly to the biological activity of quercetin than the parent compound, however, degradation products appear inactive.

## 1. Introduction

Flavonoids are secondary metabolites extensively found in many plant components, including fruits and vegetables. To date, more than 10,000 flavonoid compounds have been isolated and identified, and over 30,000 publications annually have addressed the health-promoting effects of flavonoids in recent years. Foods rich in flavonoids are often called superfoods because of their remarkable physiological benefits. They are categorized into different groups based on their chemical structure, including the degree of unsaturation and oxidation of the carbon ring. These classes encompass flavones, flavanones, isoflavones, flavonols, chalcones, and anthocyanins [1,2].

Quercetin, as a flavonol, is highly abundant in the human diet, comprising 75% of the daily consumed flavonoids [3]. Due to its wide range of pharmacological properties and its low toxicity, quercetin has been the focus of a substantial number of research studies. Pre-clinical and clinical investigations point out that quercetin has antioxidant [4], anti-inflammatory [5], anti-cancer [6], antimicrobial [7], cardioprotective, anti-diabetic, anti-obesity [8], and anti-allergic [9] properties.

In plants, quercetin occurs mainly in a glycosidic form, and the current understanding of quercetin glucoside absorption suggests that these compounds undergo enzymatic deglycosylation in the gastrointestinal tract, mostly by the activity of luminal lactase phlorizin hydrolase or cytosolic β-glucosidase within enterocytes. Following the removal of the sugar moiety, Phase II enzymes modify the aglycone through methylation, glucuronidation, and sulfation, resulting in the formation of conjugated metabolites in hepatocytes and, to a lesser extent, also in enterocytes. Unabsorbed quercetin undergoes ring cleavage by gut microbiota, producing low-molecular-weight phenolic acids that can be subsequently absorbed [10]. Previous studies indicate that the plasma concentration of the mere aglycone is hardly detectable after quercetin intake [11], as quercetin is rapidly cleared from plasma and cells and is subject to substantial first-pass metabolism [12,13]. This fact casts doubt on the activity of quercetin and leads us to question the source of activity. Thus, in the present study, we focused on the anti-inflammatory activity of the well-identified metabolites of quercetin. We studied 27 metabolites, including quercetin, and tested them in silico for their binding to ten different inflammation-related proteins. We then conducted microscale thermophoresis as an in vitro assay of the chosen metabolites to validate the in silico docking results.

## 2. Results

### 2.1. Molecular Docking

**Figure 1:**
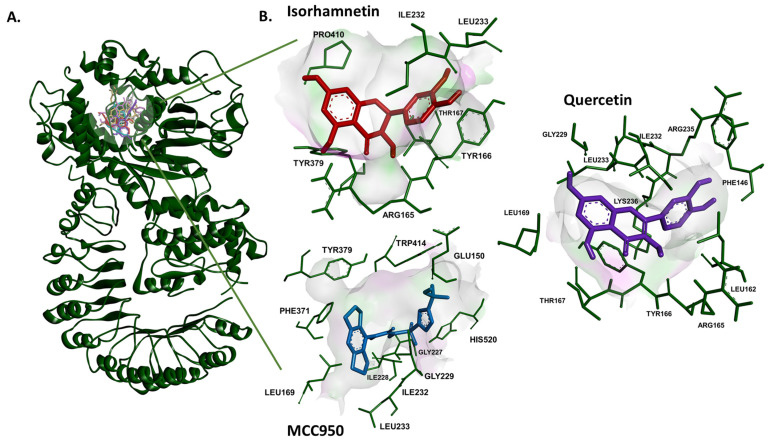
Molecular docking analysis. (**A**) The tested compounds (quercetin, quercetin 3-*O*-glucuronide, quercetin 7-*O*-glucuronide, isorhamnetin, isorhamnetin 3-*O*-glucuronide, and the positive control (MCC950) were inside the binding site of the NLRP3 (pdb:6npy) protein. (**B**) Quercetin, isorhamnetin, and MCC950 with the interacting amino acids.

The docking scores of the metabolites to ten inflammation-related proteins are shown in Table 1. In general, Phase II metabolites (glucuronides and sulfates) gave better results than phenolic acid metabolites formed by gut microorganisms. Top compounds changed depending on the protein; for NLRP3, NF-κB, IL-6R, TNF-α, IL-1β, and IL1R, quercetin 7-*O*-glucuronide-4′-*O*-sulfate and isorhamnetin 7-*O*-glucuronide-4′-*O*-sulfate were the lead compounds, whereas for COX-1, quercetin 4′-*O*-glucuronide and quercetin 3-*O*-glucuronide were at the fore. Quercetin 4′-*O*-glucuronide was also pioneering for TNFR1 and IL-6 with isorhamnetin 7-*O*-glucuronide-4′-*O*-sulfate and tamarixetin 7-*O*-glucuronide, respectively. COX-2 gave the top binding for isorhamnetin 3-*O*-glucuronide and quercetin 7-*O*-glucuronide-4′-*O*-sulfate. Benzoic acid, phloroglucinol, and 3,4-dihydroxytoluene were the last three compounds with the least binding scores for all tested proteins. The binding sites, amino acid residues, and binding scores of NLRP3 to quercetin, isorhametin, and the positive control (MCC950) are shown in Figure 1 and Table 2. To further validate the docking setup for NLRP3 as the main target, the co-crystallized ligand ADP was redocked into its binding pocket. The results are shown in Table 2 together with interactions of the co-crystallized ADP as a reference. Notably, all main interactions observed in the crystallographic ADP complex were retained in the redocked pose, despite an RMSD value of 3.90 Å. This suggests partial geometric change but substantial conservation of the binding interaction pattern and supports the protocol as a screening-oriented approach for identifying binding interactions.

### 2.2. Microscale Thermophoresis

**Figure 2:**
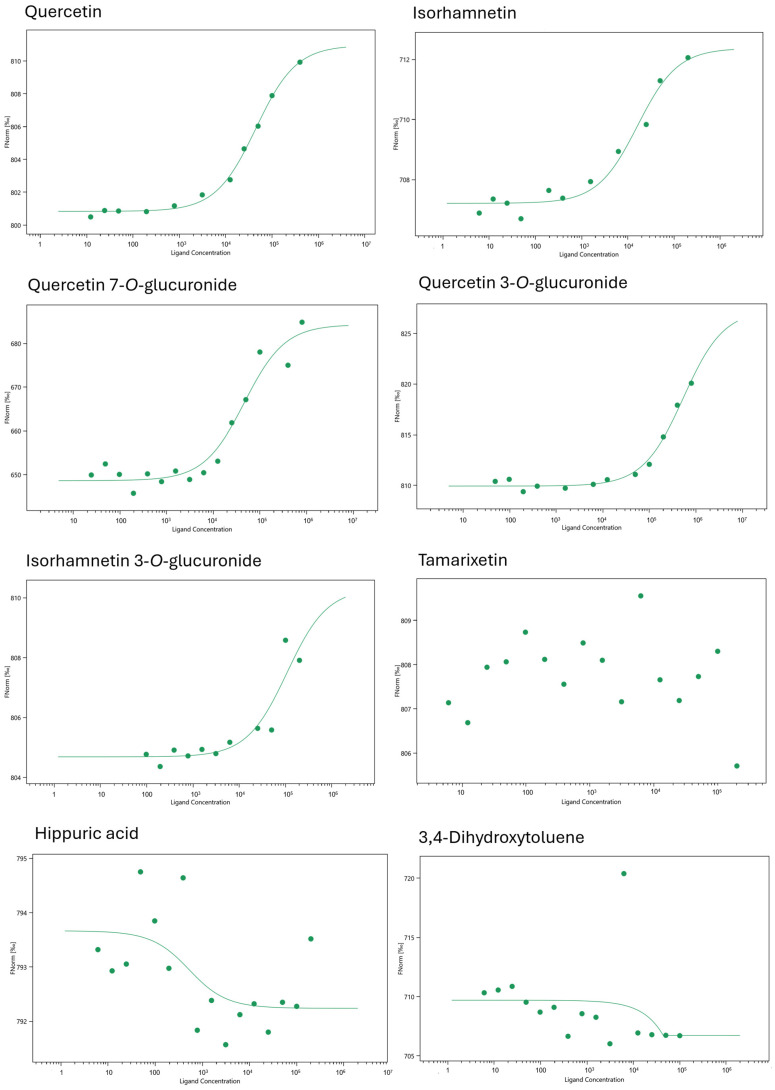
Binding of quercetin, isorhamnetin, quercetin 7-*O*-glucuronide, quercetin 3-*O*-glucuronide, isorhamnetin 3-*O*-glucuronide, tamarixetin, hippuric acid, and 3,4-dihydroxytoluene to NLRP3 as determined by microscale thermophoresis.

The binding of the compounds quercetin, quercetin 7-*O*-glucuronide, quercetin 3-*O*-glucuronide, isorhamnetin 3-*O*-glucuronide, tamarixetin, isorhamnetin, hippuric acid, and 3,4-dihydroxytoluene was tested for NLRP3 to verify the docking results. Quercetin was found to bind with a K_D_ value of 44.84 ± 4.21 µM, whereas isorhamnetin bound with 16.12 ± 5.16 µM. Glucuronides exhibited lower K_D_ values: isorhamnetin 3-*O*-glucuronide with 106.20 ± 83.15 µM, quercetin 7-*O*-glucuronide with 120.11 ± 29.09 µM, and quercetin 3-*O*-glucuronide with 536.88 ± 139.04 µM. Tamarixetin, by contrast, did not exert any binding. As anticipated, hippuric acid and 3,4-dihydroxytoluene did not display a binding curve, indicating no binding to NLRP3. Since docking analysis of both compounds gave relatively low binding energy scores, MST confirmed the results of the in silico analysis of these compounds (Figure 2).

## 3. Discussion

Quercetin is the most intensely studied flavonoid in plant-based foods. It is primarily present as quercetin glycosides in food sources. Following consumption, absorption takes place in the intestines by a sodium-dependent glucose co-transporter (SGLT), then it is deglycosylated to the aglycone, quercetin, by cytoplasmic β-glucosidase (CBG). Another route implies the deglycosylation of quercetin by lactase-rhizosphingoside hydrolase, then absorption by passive diffusion by intestinal epithelial cells. Uridine diphosphate glucuronosyltransferases (UGTs) are responsible for the glucuronidation of quercetin, and catechol-*O*-methyltransferases (COMTs) for *O*-methylation in the small intestine. Metabolization continues in the liver by *O*-methylation, mainly leading to isorhamnetin (3′-*O*-methylquercetin) and, to a smaller extent, to tamarixetin (4′-*O*-methylquercetin), followed by sulfation at the oxygens at positions 7 and 3′, and glucuronidation at the positions 3, 7, 3′, and 4′. Quercetin conjugates from the liver and the small intestine, or unmetabolized quercetin, encounter the colon bacteria in the gut, being degraded to diverse phenolic acids. The metabolic cleavage of quercetin and related compounds occurs mainly by fission of the heterocyclic C-ring. This leads, for the still intact B-ring part, i.e., to the formation of 3,4-dihydroxyphenylacetic acid (DOPAC), 3-hydroxyphenylacetic acid (3-OPAC), and 3,4-dihydroxybenzoic acid, while the ring A fragment yields, i.e., phloroglucinol and 2,4,6-trihydroxybenzoic acid. Other low-molecular-weight phenolic compounds, like vanillic acid, homovanillic acid, 3,4-dihydroxytoluene, 3-methoxy-4-hydroxyphenylacetic acid, as well as 3-(3′,4′-dihydroxyphenyl) propionic acid and 3-(3′-hydroxyphenyl) propionic acid, were also detected. If absorbed, these conjugates are transferred to the liver and eliminated in the urine; unabsorbed conjugates are eliminated through the feces [14,15].

In this study, we formed a 27-compound mini-library with well-known quercetin metabolites and degradation products, including quercetin as the parent compound. Molecular docking of the library was performed against ten different inflammation-related proteins, including NLRP3, NF-κB, IL-6, COX-1, COX-2, TNF-α, TNFR1, IL-6R, IL-1β, and IL-1R1. Those proteins were selected as they are crucial, interconnected components of the inflammatory signaling network, covering multiple levels of the inflammatory cascade, including upstream sensor (NLRP3), transcription factor (NF-κB), pro-inflammatory cytokines (TNF-α, IL-6, IL-1β), their receptors (TNFR1, IL-6R, IL-1R), and downstream effector enzymes (COX-1/2). Binding scores of the docking analysis revealed that glucuronides or sulfated metabolites had better binding affinities compared to *O*-methylated metabolites and degradation products. The reason for this may be that larger molecules usually have more functional groups, leading to multiple interactions, and having more rotatable bonds, resulting in higher flexibility and conformational adaptability, and occupation of more area in the binding pocket, resulting in better shape complementarity. Therefore, we wanted to confirm our in silico results by conducting microscale thermophoresis as an in vitro binding assay. For this purpose, we selected the NLRP3 protein because it has unique importance due to its broad sensing capability, pivotal function in inflammatory signaling, and involvement in a wide range of diseases. It stands out from other inflammatory proteins due to its ability to integrate various danger signals and to drive pivotal inflammatory pathways, making it a top target for investigation and treatment [16,17,18]. Since it has been documented that in humans, the major circulating plasma metabolites were quercetin 3′-*O*-sulfate, quercetin 3-*O*-glucuronide, and isorhamnetin 3-*O*-glucuronide [19], the commercially available quercetin 3-*O*-glucuronide and isorhamnetin 3-*O*-glucuronide were initially chosen, along with quercetin 7-*O*-glucuronide. Then, two *O*-methylated metabolites, isorhamnetin and tamarixetin, were picked for comparison, as they ranked in the middle of the docking results. In addition, two degradation products, hippuric acid and 3,4-dihydroxytoluene, were selected from the least active compounds based on the docking results. As anticipated, the two, quite small degradation products did not show any binding, thus confirming the corresponding in silico results. In all the MST assays, isorhamnetin exhibited the lowest binding energy and the highest binding affinity, followed by quercetin. Going back to the molecular docking results, Ile232, Leu233, and Tyr379 were shared amino acid residues by both isorhamnetin and the positive control (MCC90). Ile232 and Leu233 were also shared binding residues for both quercetin and MCC950, along with Leu169 and Gly229.

The comparison of the different metabolites of quercetin on various activities in the literature has been done previously. A previous in silico study revealed that molecular docking of quercetin towards NLRP3 protein yielded a LBE of −5.3 kcal/mol, while our docking result showed a stronger binding affinity (LBE = −8.5 kcal/mol) [20]. Furthermore, Boreak et al. screened a small library of 329 plant-derived natural compounds against human IL-1β and selected the top five hits based on binding affinity and docking scores. Among these, quercetin showed the highest binding affinity (−10.3 kcal/mol) and the most favorable interactions with IL-1β compared to the other four compounds [21]. In our study, quercetin docking with IL-1β showed a lower binding affinity with LBE equal to −7.25 kcal/mol. In addition to that, the molecular docking of the metabolite isorhamnetin against COX-2 yielded a score of −8.5 kcal/mol [22], whereas our result was −9.31 kcal/mol. The docking of isorhamnetin to the transcription factor NF-κB gave a score of −7.94 kcal/mol [23], while our docking score was −6.75 kcal/mol. Comparing our results with published ones showed a minimal variation in LBE scores, and this could be for several reasons, such as working with different PDB IDs, different docking programs, and different running parameters. The quercetin sulfated metabolites were experimented with quercetin and are usually less active as radical scavengers, reducing, or anti-lipoperoxidant agents than quercetin itself [24]. The activity of quercetin has been compared with that of isorhamnetin, tamarixetin, quercetin 3′-*O*-sulfate, and quercetin 7-*O*-sulfate on COX-2 inhibition. Isorhamnetin and tamarixetin were potent inhibitors, each reducing COX-2 levels by >90%, while the parent compound, quercetin itself, was less effective, causing only a 50% reduction, and quercetin 3- and 7-*O*-sulfate had no effect on COX-2 levels [25]. In another study, quercetin, isorhamnetin, and quercetin 3-glucuronide were investigated for their inflammatory gene expression. Quercetin and isorhamnetin significantly decreased mRNA and protein levels of iNOS as well as mRNA levels of IL1β, IL6, and MIP1α in LPS-stimulated macrophages, while quercetin 3-glucuronide did not alter these pro-inflammatory markers. NF-κB activity was inhibited by quercetin and isorhamnetin but not by quercetin3-glucuronide. Proinflammatory microRNA-155 was down-regulated by quercetin and isorhamnetin but not by 3-glucuronide metabolite [26]. Different mechanisms have been reported to explain the anti-inflammatory activity of isorhamnetin. It had an inhibitory effect on NLRP3 and AIM2 inflammasomes and down-regulatory activity on the expression of pro-inflammatory cytokines in vitro [27]. In vivo, isorhamnetin, at a dose of 100 mg/kg, significantly decreased the hepatic levels of NF-κB, NLRP3, caspase 1, IL-6, TNFα, MPO, TBARS, and ROS, in addition to serum levels of ALT, ALP, and AST. It also prevented the decline of GSH, SOD activity, Sirtuin 1, and Nrf2 in acetaminophen-induced liver injury in mice [28]. COX-2 expression inhibition was observed in mice with LPS-induced acute lung injury by isorhamnetin at a dose of 60 mg/kg [29]. Isorhamnetin reduced LPS-induced transcriptional and protein levels of pro-IL-1β, TNF-α, IL-6, and NLRP3 itself, suggesting that isorhamnetin suppressed NF-κB signaling or upstream events in the priming phase of NLRP3. In the activation step, isorhamnetin inhibited IL-1β and IL-18 secretion and active caspase-1 (p20) generation. Direct enzymatic inhibition of active recombinant caspase-1 was also observed by isorhamnetin [27]. To the best of our knowledge, direct binding of isorhamnetin to NLRP3 has not been demonstrated so far.

In our study, we did not find selected quercetin degradation products to be active in silico or in vitro, while some previous studies suggested that quercetin degradation products possess biological activity across various pathways. Tang et al. (2016) compared the in vitro activity of quercetin, DOPAC, 3-hydroxyphenylacetic acid (OPAC), 3,4-dihydroxybenzoic acid (PCA), and hippuric acid on DPPH radicals, superoxide, and detoxification enzyme levels [30]. They found that OPAC and hippuric acid showed no scavenging activity, while DOPAC presented similar activity as quercetin and higher activity than PCA. The superoxide dismutase-like activity of DOPAC was significantly lower than that of quercetin but higher than that of PCA. Again, OPAC and hippuric acid showed no activity at the tested concentration of 50 μM. Quercetin, Rutin, DOPAC, OPAC, and homovanillic acid were compared by means of antioxidant, reducing, and chelating activity, plus their ability to inhibit Advanced Glycation End-products (AGEs) formation, which is an indicator of aging, oxidative stress, chronic inflammation, metabolic dysfunction, and cardiovascular risk. Quercetin showed the highest antioxidant and reducing activity, while rutin demonstrated the best chelating activity (85%), followed by quercetin (72%). Inhibition of AGEs by rutin and quercetin was high and comparable, followed by DOPAC [31]. Olthof et al. (2003) suggested that the metabolites of dietary phenols have less antioxidant activity compared to their parent compounds [32]. Hence, the in vivo contribution of dietary phenols to antioxidant activity may be less than anticipated based on in vitro assessments. Protective effects of quercetin (10 µM) and protocatechuic acid (40 µM) on intestinal porcine epithelial (IPEC-1) cells infected with enterotoxigenic *E. coli* (ETEC K88) were investigated. Quercetin more effectively inhibited CD14, TLR4, IRAK1, and NF-κB than PCA and was stronger in reducing TNF-α and IL-6. Necroptosis and pyroptosis pathways, including NLRP3 and caspase-1, were also inhibited similarly by both quercetin and PCA. However, overall, quercetin was more potent considering the lower dosage (40 vs. 10 µM) [33].

The initial objective of this study leads to another question. It is known that quercetin is mostly metabolized to its glucuronides, resulting in hardly detectable plasma concentrations of the aglycone. Isorhamnetin and its glucuronide were also detected, but usually in smaller quantities. Thus, if the glucuronides are not as active as the aglycone, quercetin itself, and if also the degradation products are not particularly active, the question arises of what is actually causing the well-known activity of quercetin. Some studies pointed out that β-glucuronidase enzymes released by immune cells can deconjugate quercetin glucuronides at the target sites, with renewed formation of the active aglycone locally, which would regain its biological activity exactly at the site where it is needed. In this case, quercetin glucuronides may serve as site-specific reservoirs, like prodrugs. Quercetin reduced the inflammation and tissue damage in a range of illness situations by blocking the activation of the NLRP3 inflammasome in both cell and animal models [34]

## 4. Materials and Methods

### 4.1. Quercetin Metabolite Library

**Figure 3:**
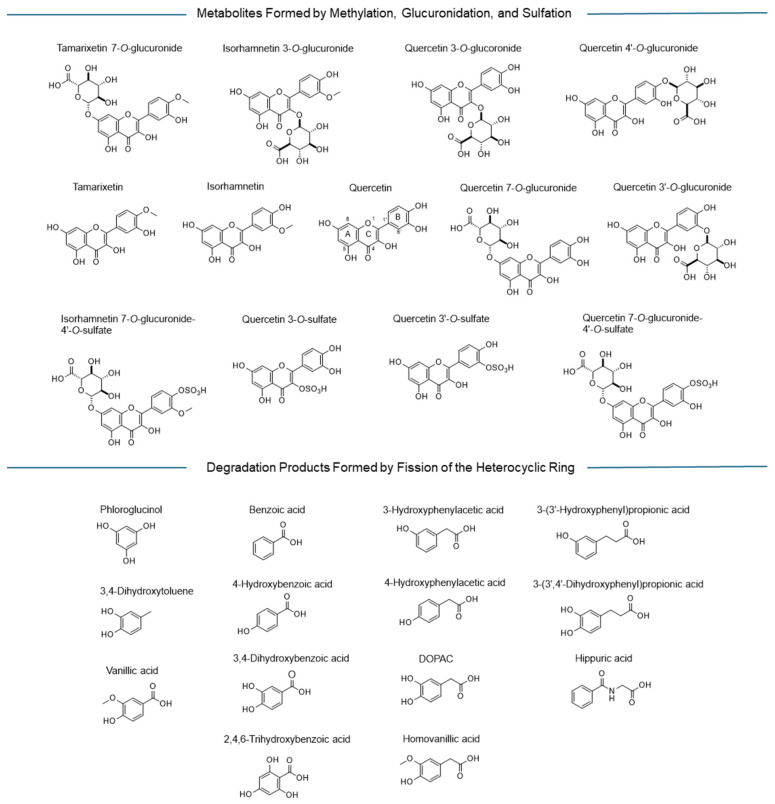
The compounds of the quercetin metabolite library were compiled through literature mining. Exemplarily for the parent compound quercetin, the rings A, B, and C are defined, as also the most important position numbers, relevant for the different derivatives.

A library of quercetin metabolites was formed with 27 compounds, including quercetin itself. The compounds were quercetin, quercetin 3-*O*-glucuronide, quercetin 7-*O*-glucuronide, quercetin 3′-*O*-glucuronide, quercetin 4′-*O*-glucuronide, quercetin 3-*O*-sulfate, quercetin 3′-*O*-sulfate, isorhamnetin, isorhamnetin 3-*O*-glucuronide, tamarixetin [35], isorhamnetin 7-*O*-glucuronide-4′-*O*-sulfate, quercetin 7-*O*-glucuronide-4′-*O*-sulfate [36], tamarixetin 7-*O*-glucuronide [37], DOPAC (3,4-dihydroxyphenylacetic acid), 3,4-dihydroxybenzoic acid (protocatechuic acid), homovanillic acid [38], 3-(3′,4′-dihydroxyphenyl) propionic acid, 3-(3′-hydroxyphenyl) propionic acid, phloroglucinol [39], 3-hydroxyphenylacetic acid, 4-hydroxyphenylacetic acid, 3,4-dihydroxytoluene, vanillic acid (3-methoxy-4-hydroxybenzoic acid), 2,4,6-trihydroxybenzoic acid, benzoic acid, hippuric acid, and 4-hydroxybenzoic acid [35,37,40] (Figure 3).

### 4.2. Docking of the Library Compounds

The protein PDB files NLRP3 (PDB: 6npy), NF-κB (PDB: 1a3q), COX-1 (PDB: 6y3c), COX-2 (PDB: 5f19), TNF-α (PDB: 2az5), TNFR1 (PDB: 1ext), IL-6 (PDB: 1alu), IL-6R (PDB: 1n26), IL-1β (PDB: 5r8q), and IL-1R (PDB: 1itb), were downloaded from the RCSB protein data bank (rcsb.org, accessed on 7 November 2023). The proteins were prepared using AutoDock 1.5.6 by adding polar hydrogen atoms, repairing missing atoms, and adding Kollman charges. Binding sites were determined based on active sites from the literature, and the CASTp 3.0 program was used for both verification and additional information. The amino acid residues were included in a grid box. The docking coordinates are shown in Table S1. The Lamarckian algorithm was applied with 250 runs and 2,500,000 energy evaluations. The library compounds were downloaded as 3D SDF files from the PubChem database (pubchem.ncbi.nlm.nih.gov, accessed on 1 June 2021) and converted to PDB files by Chem3D (revvitysignals.com/products/research/chemdraw, accessed on 11 June 2021) with energy minimization to acquire optimized geometries and, later on, converted to PDBQT files by PyRx (pyrx.sourceforge.io/, accessed on 11 June 2021). In this step, polar hydrogens and Gasteiger partial charges were added automatically to guarantee compatibility with AutoDock. Molecular docking was conducted using the supercomputer MOGON and advisory services offered by Johannes Gutenberg University Mainz (hpc.uni-mainz.de), which is a member of the AHRP (Alliance for High-Performance Computing in Rhineland-Palatinate, www.ahrp.info, accessed on 21 December 2023). AutoDock generated dlg files which were used to acquire the lowest binding energies (LBEs) along with their anticipated inhibition constants (pK_i_ values). Using Discovery Studio Visualizer (discover.3ds.com/discovery-studio-visualizer-download, accessed on 1 June 2025), the conformations corresponding to the LBEs were visualized, and interacting amino acids were identified from each compound’s 2D diagram. For Root Mean Square Deviation (RMSD) calculation of the ADP redocking, VMD version 1.9.4a53 was used [41]. Therefore, the crystallographic and redocked complexes were superposed based on the protein backbone, and the RMSD was calculated using ligand heavy atoms.

### 4.3. Microscale Thermophoresis Assay

Microscale thermophoresis (MST) was conducted to confirm the in silico binding data exemplarily with selected quercetin metabolites. Recombinant NLRP3 (Origene, TP750176) was labeled with the Monolith Protein Labeling Kit RED-NHS 2nd Generation (Cat. No. M0-L011, NanoTemper Technologies, Munich, Germany). In MST buffer (50 mM Tris base, 150 mM NaCl, 10 mM MgCl_2_, and 0.05% *v*/*v* Tween 20, pH 7.4), the compounds were prepared in 16 concentrations, ranging from 400 μM down to 12.2 nM, with a constant DMSO ratio of 2%. A Nanodrop 1000 instrument (PEQLAB, Erlangen, Germany) was used to measure the concentration of labeled NLRP3 (c = 180 nM). The labeled proteins were added as 1:1 dilutions, thus reducing the concentration of the compounds stepwise by half from 200 μM down to 6.1 nM. The tested compounds, quercetin 3-*O*-glucuronide, quercetin 7-*O*-glucuronide, isorhamnetin, isorhamnetin 3-*O*-glucuronide, and tamarixetin, were supplied from Fischer Analytics GmbH (Bingen am Rhein, Germany), while hippuric acid and 3,4-dihydroxytoluene were obtained from Merck (Darmstadt, Germany) (112003 and M34200). The samples were measured utilizing Monolith NT.115 (Nano Temper Technologies, Munich, Germany). The MST power was set to 10%, 20%, 40%, 60%, and 80%, and the light-emitting diodes (LED) power was 20–40%, depending on the fluorescence of the aliquots at 20 °C. The fitting curves with K_D_ values were calculated with MO. Affinity analysis software v2.2.4 (NanoTemper Technologies).

## 5. Conclusions

In this study, we established a small library of quercetin metabolites containing 26 metabolites plus quercetin itself by mining the literature. We tested the compounds towards ten inflammatory target proteins, including NLRP3, NF-κβ, COX-1, COX-2, TNF-α, TNFR1, IL-6, IL-6R, IL-1β, and IL-1R in silico. We then chose the NLRP3 inflammasome protein and quercetin 3- and 7-*O*-glucuronides, isorhamnetin 3-*O*-glucuronide, tamarixetin, isorhamnetin, hippuric acid, and 3,4-hydroxytoluene from the metabolites to conduct a binding assay by MST in vitro. Among the investigated compounds, isorhamnetin gave the best binding value, even better than quercetin itself. Glucuronides also bind, but only at higher concentrations. Degraded metabolites (like hippuric acid and 3,4-hydroxytoluene) did not bind to the NLRP3 protein.

## Figures and Tables

**Table 1:** Docking results of the quercetin metabolites towards ten proteins. The LBE [kcal/mol] and pKi [nM] values of the quercetin metabolites with the highest affinities to their corresponding proteins were highlighted in bold.

**Compounds**	**NLRP3**			**COX-1**			**COX-2**	
	**LBE [kcal/mol]**		**pKi [nM]**	**LBE [kcal/mol]**	**pKi [nM]**		**LBE [kcal/mol]**	**pKi [nM]**
Quercetin 7-*O*-glucuronide-4′-*O*-sulfate	**−12.76 ± 0.15**		**0.46 ± 0.12**	−11.90 ± 0.19	1.99 ± 0.67		**−13.71 ± 0.00**	**(89.48 ± 0.00) × 10^−3^**
Isorhamnetin 7-*O*-glucuronide-4′-*O*-sulfate	**−12.19 ± 0.26**		**1.22 ± 0.46**	−11.85 ± 0.37	2.41 ± 1.10		−12.72 ± 0.18	0.50 ± 0.143
Quercetin 3-*O*-glucuronide	−11.10 ± 0.09		7.33 ± 1.14	−10.86 ± 0.46	13.91 ± 7.84		−11.38 ± 0.32	5.27 ± 2.64
Isorhamnetin 3-*O*-glucuronide	−10.88 ± 0.49		13.22 ± 11.04	−10.59 ± 0.46	22.07 ± 11.65		**−15.24 ± 0.00**	**(6.79 ± 0.07) × 10^−3^**
Quercetin 4′-*O*-glucuronide	−10.80 ± 0.15		12.40 ± 2.90	**−13.33 ± 0.25**	**0.19 ± 0.08**		−12.88 ± 0.18	0.38 ± 0.11
Quercetin 7-*O*-glucuronide	−10.63 ± 0.11		16.29 ± 3.19	**−13.05 ± 0.20**	**0.29 ± 0.09**		−13.36 ± 0.15	0.17 ± 0.04
Tamarixetin 7-*O*-glucuronide	−10.46 ± 0.28		23.04 ± 9.17	−12.01 ± 0.28	1.77 ± 0.88		−12.47 ± 0.00	0.72 ± 0.00
Quercetin 3′-*O*-glucuronide	−10.08 ± 0.05		40.95 ± 3.73	−12.59 ± 0.27	0.65 ± 0.30		−13.45 ± 0.27	0.15 ± 0.06
Quercetin 3′-*O*-sulfate	−9.80 ± 0.22		68.99 ± 25.13	−12.69 ± 0.11	0.50 ± 0.10		−11.85 ± 0.04	2.06 ± 0.14
Quercetin 3-*O*-sulfate	−9.59 ± 0.11		93.92 ± 13.88	−11.79 ± 0.08	2.32 ± 0.29		−11.25 ± 0.02	5.65 ± 0.23
Isorhamnetin	−9.03 ± 0.03		239.37 ± 9.06	−9.37 ± 0.06	135.22 ± 13.18		−9.31 ± 0.08	151.17 ± 20.57
Quercetin	−8.54 ± 0.34		595.34 ± 290.82	−9.75 ± 0.02	70.83 ± 1.94		−9.97 ± 0.00	48.89 ± 0.41
Tamarixetin	−8.49 ± 0.01		598.26 ± 12.84	−9.41 ± 0.12	78.04 ± 58.02		−9.44 ± 0.01	119.74 ± 2.70
3-(3′,4′-Dihydroxyphenyl) propanoic acid	−7.54 ± 0.01		(2.96 ± 0.02) × 10^3^	−7.89 ± 0.28	(1.85 ± 0.94) × 10^3^		−7.91 ± 0.13	(1.64 ± 0.34) × 10^3^
3-(3′-Hydroxyphenyl) propanoic acid	−7.00 ± 0.01		(7.37 ± 0.17) × 10^3^	−7.59 ± 0.01	(2.75 ± 0.05) × 10^3^		−7.34 ± 0.10	(4.22 ± 0.65) × 10^3^
DOPAC	−6.80 ± 0.04		(10.44 ± 0.66) × 10^3^	−7.37 ± 0.01	(3.96 ± 0.07) × 10^3^		−7.26 ± 0.06	(4.77 ± 0.45) × 10^3^
Hippuric acid	−6.73 ± 0.01		(11.77 ± 0.04) × 10^3^	−6.57 ± 0.14	15.69 ± 3.83) × 10^3^		−6.96 ± 0.03	(7.98 ± 0.38) × 10^3^
Homovanillic acid	−6.66 ± 0.03		(13.25 ± 0.66) × 10^3^	−7.56 ± 0.00	(2.85 ± 0.00) × 10^3^		−7.13 ± 0.01	(5.93 ± 0.08) × 10^3^
3-Hydroxyphenylacetic acid	−6.33 ± 0.00		(22.83 ± 0.08) × 10^3^	−6.98 ± 0.00	(7.60 ± 0.03) × 10^3^		−6.92 ± 0.02	(8.49 ± 0.33) × 10^3^
Vanillic acid	−6.15 ± 0.01		(30.82 ± 0.09) × 10^3^	−6.61 ± 0.00	(14.32 ± 0.07) × 10^3^		−6.86 ± 0.00	(9.38 ± 0.03) × 10^3^
4-Hydroxyphenylacetic acid	−6.03 ± 0.02		(37.66 ± 1.3) × 10^3^	−6.50 ± 0.00	(17.31 ± 0.05) × 10^3^		−6.59 ± 0.13	(15.26 ± 3.21) × 10^3^
4-Hydroxybenzoic acid	−5.96 ± 0.01		(95.04 ± 3.4) × 10^3^	−5.88 ± 0.00	(48.98 ± 0.10) × 10^3^		−6.49 ± 0.01	(16.10 ± 1.11) × 10^3^
3,4-Dihydroxybenzoic acid	−5.96 ± 0.01		(43.15 ± 0.23) × 10^3^	−6.48 ± 0.04	(17.92 ± 1.18) × 10^3^		−6.83 ± 0.00	(9.87 ± 0.08) × 10^3^
2,4,6-Trihydroxybenzoic acid	−5.41 ± 0.02		(107.63 ± 3.5) × 10^3^	−6.26 ± 0.01	(25.85 ± 0.31) × 10^3^		−6.77 ± 0.06	(10.87 ± 1.04) × 10^3^
Benzoic acid	−4.97 ± 0.01		(228.07 ± 1.2) × 10^3^	−5.52 ± 0.00	(90.06 ± 0.03) × 10^3^		−5.79 ± 0.00	(56.80 ± 0.16) × 10^3^
Phloroglucinol	−4.45 ± 0.00		(548.60 ± 0.32) × 10^3^	−5.33 ± 0.02	(121.74 ± 0.04) × 10^3^		−5.03 ± 0.02	(204.95 ± 5.28) × 10^3^
3,4-Dihydroxytoluene	−4.43 ± 0.00		(569.44 ± 0.19) × 10^3^	−5.17 ± 0.00	(161.4 ± 0.56) × 10^3^		−5.17 ± 0.03	(163.11 ± 8.27) × 10^3^
**Compounds**	**TNF-α**			**TNFR1**			**NF-κB**	
	**LBE [kcal/mol]**		**pKi [nM]**	**LBE [kcal/mol]**	**pKi [nM]**		**LBE [kcal/mol]**	**pKi [nM]**
Quercetin 7-*O*-glucuronide-4′-*O*-sulfate	**−10.78 ± 0.22**		**13.29 ± 4.94**	−12.53 ± 0.05	0.66 ± 0.06		**−11.32 ± 0.16**	**5.22 ± 1.5**
Isorhamnetin 7-*O*-glucuronide-4′-*O*-sulfate	**−10.64 ± 0.04**		**16.09 ± 1.18**	**−13.06 ± 0.54**	**0.73 ± 0.07**		**−11.44 ± 0.06**	**4.13 ± 0.41**
Quercetin 3-*O*-glucuronide	−10.47 ± 0.27		48.03 ± 5.52	−12.76 ± 0.13	0.46 ± 0.09		−8.77 ± 0.32	302.64 ± 118.94
Isorhamnetin 3-*O*-glucuronide	−10.47 ± 0.27		23.28 ± 9.88	−11.23 ± 0.04	6.10 ± 1.59		−9.14 ± 0.02	200.90 ± 4.82
Quercetin 4′-*O*-glucuronide	−9.99 ± 0.11		48.79 ± 8.71	**−13.40 ± 0.03**	**0.15 ± 0.01**		−9.32 ± 0.29	159.15 ± 75.09
Quercetin 7-*O*-glucuronide	−9.48 ± 0.12		114.50 ± 20.78	−11.28 ± 0.04	5.38 ± 0.36		−9.23 ± 0.08	171.67 ± 20.55
Tamarixetin 7-*O*-glucuronide	−9.94 ± 0.02		51.72 ± 1.78	−11.80 ± 0.03	2.24 ± 0.11		−9.26 ± 0.07	163.26 ± 20.92
Quercetin 3′-*O*-glucuronide	−9.73 ± 0.05		73.65 ± 6.30	−12.16 ± 0.02	1.21 ± 0.06		−9.09 ± 0.62	124.48 ± 40.05
Quercetin 3′-*O*-sulfate	−8.88 ± 0.01		312.33 ± 2.00	−10.04 ± 0.09	44.76 ± 9.61		−8.70 ± 0.36	602.00 ± 13.82
Quercetin 3-*O*-sulfate	−9.49 ± 0.01		111.30 ± 1.86	−10.13 ± 0.13	38.4 ± 8.23		−8.42 ± 0.03	671.72 ± 27.05
Isorhamnetin	−7.09 ± 0.08		(6.45 ± 0.81) × 10^3^	−8.02 ± 0.00	(1.34 ± 0.01) × 10^3^		−6.75 ± 0.09	(11.44 ± 1.61) × 10^3^
Quercetin	−8.53 ± 0.03		556.94 ± 33.63	−8.70 ± 0.00	421.47 ± 10.34		−5.94 ± 0.08	(41.51 ± 4.27) × 10^3^
Tamarixetin	−7.50 ± 0.01		(3.15 ± 0.04) × 10^3^	−8.73 ± 0.04	417.06 ± 32.74		−6.13 ± 0.01	(32.22 ± 0.54) × 10^3^
3-(3′,4′-dihydroxyphenyl) propanoic acid	−7.34 ± 0.01		(4.14 ± 0.10) × 10^3^	−7.13 ± 0.00	(5.92 ± 0.11) × 10^3^		−6.94 ± 0.01	(8.19 ± 0.12) × 10^3^
3-(3′-hydroxyphenyl) propanoic acid	−6.88 ± 0.00		(9.03 ± 0.03) × 10^3^	−6.37 ± 0.00	(21.31 ± 0.16) × 10^3^		−6.27 ± 0.02	(25.40 ± 0.84) × 10^3^
DOPAC	−6.82 ± 0.01		(10.10 ± 0.24) × 10^3^	−5.95 ± 0.01	(43.73 ± 5.90) × 10^3^		−6.05 ± 0.01	(36.94 ± 0.35) × 10^3^
Hippuric acid	−5.88 ± 0.00		(48.85 ± 0.40) × 10^3^	−6.16 ± 0.00	(30.53 ± 0.24) × 10^3^		−6.73 ± 0.01	(11.77 ± 0.06) × 10^3^
Homovanillic acid	−6.47 ± 0.00		(17.89 ± 0.17) × 10^3^	−6.22 ± 0.01	(27.55 ± 0.34) × 10^3^		−6.33 ± 0.01	(22.74 ± 0.10) × 10^3^
3-Hydroxyphenylacetic acid	−6.19 ± 0.00		(28.86 ± 0.20) × 10^3^	−5.65 ± 0.00	(71.66 ± 1.01) × 10^3^		−5.83 ± 0.08	(53.38 ± 6.83) × 10^3^
Vanillic acid	−5.12 ± 0.00		(177.69 ± 0.41) × 10^3^	−5.32 ± 0.02	(125.92 ± 4.80) × 10^3^		−5.30 ± 0.01	(129.26 ± 2.03) × 10^3^
4-Hydroxyphenylacetic acid	−5.67 ± 0.00		(69.14 ± 0.58) × 10^3^	−5.44 ± 0.01	(103.51 ± 0.61) × 10^3^		−5.70 ± 0.00	(66.22 ± 0.50) × 10^3^
4-Hydroxybenzoic acid	−4.6 ± 0.00		(426.46 ± 1.54) × 10^3^	−4.91 ± 0.01	(251.04 ± 4.86) × 10^3^		−4.77 ± 0.01	(317.34 ± 4.75) × 10^3^
3,4-Dihydroxybenzoic acid	−5.29 ± 0.00		(131.77 ± 0.17) × 10^3^	−5.22 ± 0.00	(149.23 ± 0.77) × 10^3^		−5.36 ± 0.01	(116.96 ± 0.46) × 10^3^
2,4,6-Trihydroxybenzoic acid	−5.87 ± 0.12		(51.11 ± 9.36) × 10^3^	−5.07 ± 0.01	(191.52 ± 2.87) × 10^3^		−5.81 ± 0.01	(54.72 ± 0.76) × 10^3^
Benzoic acid	−4.14 ± 0.00		(926.23 ± 0.79) × 10^3^	−4.54 ± 0.00	(467.51 ± 1.54) × 10^3^		−4.24 ± 0.01	(770.96 ± 8.71) × 10^3^
Phloroglucinol	−4.12 ± 0.00		(957.62 ± 3.62) × 10^3^	−4.15 ± 0.01	(907.7 ± 13.54) × 10^3^		−3.75 ± 0.01	(1773.33 ± 11.54) × 10^3^
3,4-Dihydroxytoluene	−4.32 ± 0.01		(678.90 ± 12.04) × 10^3^	−4.09 ± 0.00	(1006.67 ± 4.71) × 10^3^		−3.90 ± 0.00	(1383.33 ± 5.77) × 10^3^
**Compounds**	**IL-6**		**IL-6R**		**IL-1β**		**IL-1R**	
	**LBE [kcal/mol]**	**pKi [nM]**	**LBE [kcal/mol]**	**pKi [nM]**	**LBE [kcal/mol]**	**pKi [nM]**	**LBE [kcal/mol]**	**pKi [nM]**
Quercetin 7-*O*-glucuronide-4′-*O*-sulfate	−8.18 ± 0.01	(1.03 ± 0.00) × 10^3^	**−9.64 ± 0.04**	**86.66 ± 5.93**	**−11.40 ± 0.06**	**4.44 ± 0.47**	−9.27 ± 0.00	159.62 ± 0.00
Isorhamnetin 7-*O*-glucuronide-4′-*O*-sulfate	−8.59 ± 0.14	515.28 ± 127.88	**−9.57 ± 0.03**	**97.60 ± 3.67**	**−10.49 ± 0.05**	**20.49 ± 1.57**	**−9.84 ± 0.00**	**62.12 ± 0.63**
Quercetin 3-*O*-glucuronide	−8.47 ± 0.03	616.86 ± 35.91	−8.07 ± 0.07	(1.25 ± 0.13) × 10^3^	−9.56 ± 0.01	98.02 ± 1.51	−7.70 ± 0.17	(2.37 ± 0.67) × 10^3^
Isorhamnetin 3-*O*-glucuronide	−8.8 ± 0.03	351.44 ± 18.86	−8.95 ± 0.13	285.76 ± 59.33	−10.33 ± 0.01	26.53 ± 0.46	−8.94 ± 0.16	293.87 ± 75.41
Quercetin 4′-*O*-glucuronide	**−9.32 ± 0.08**	**149.25 ± 18.92**	−8.76 ± 0.02	380.87 ± 10.56	−9.83 ± 0.05	62.87 ± 4.62	−9.19 ± 0.00	181.98 ± 0.00
Quercetin 7-*O*-glucuronide	−7.92 ± 0.02	(1.66 ± 0.13) × 10^3^	−8.79 ± 0.01	358.95 ± 4.56	−9.15 ± 0.02	197.28 ± 6.72	−8.78 ± 0.00	369.70 ± 0.12
Tamarixetin 7-*O*-glucuronide	**−9.43 ± 0.01**	**122.45 ± 2.64**	−8.39 ± 0.05	708.61 ± 58.10	−9.18 ± 0.01	186.73 ± 3.76	−8.94 ± 0.00	279.50 ± 0.00
Quercetin 3′-*O*-glucuronide	−7.78 ± 0.04	(2.01 ± 0.17) × 10^3^	−8.67 ± 0.01	441.23 ± 7.78	−9.36 ± 0.09	141.11 ± 22.75	**−9.32 ± 0.00**	**147.94 ± 0.00**
Quercetin 3′-*O*-sulfate	−7.28 ± 0.17	(4.84 ± 1.26) × 10^3^	−7.82 ± 0.13	(1.89 ± 0.39) × 10^3^	−8.57 ± 0.00	527.45 ± 7.69	−7.66 ± 0.17	(2.54 ± 0.69) × 10^3^
Quercetin 3-*O*-sulfate	−7.61 ± 0.00	(2.67 ± 0.01) × 10^3^	−7.91 ± 0.01	(1.59 ± 0.02) × 10^3^	−7.38 ± 0.01	(3.86 ± 0.07) × 10^3^	−7.98 ± 0.04	(1.42 ± 0.11) × 10^3^
Isorhamnetin	−6.46 ± 0.00	(18.45 ± 0.02) × 10^3^	−6.39 ± 0.02	(20.73 ± 0.81) × 10^3^	−5.90 ± 0.00	(47.53 ± 0.13) × 10^3^	−7.15 ± 0.02	(5.77 ± 0.24) × 10^3^
Quercetin	−6.98 ± 0.14	(7.86 ± 1.69) × 10^3^×	−6.77 ± 0.00	(10.94 ± 0.05) × 10^3^	−7.25 ± 0.02	(4.91 ± 0.2) × 10^3^	−7.07 ± 0.01	(6.61 ± 0.03) × 10^3^
Tamarixetin	−6.23 ± 0.21	(28.98 ± 9.32) × 10^3^	−6.45 ± 0.00	(18.65 ± 0.01) × 10^3^	−7.56 ± 0.08	(2.92 ± 0.40) × 10^3^	−7.10 ± 0.06	(6.29 ± 0.61) × 10^3^
3-(3′,4′-Dihydroxyphenyl) propanoic acid	−6.61 ± 0.00	(14.2 ± 0.16) × 10^3^	−6.39 ± 0.01	(20.79 ± 0.40) × 10^3^	−7.89 ± 0.04	(1.64 ± 0.12) × 10^3^	−6.09 ± 0.03	(34.39 ± 1.73) × 10^3^
3-(3′-Hydroxyphenyl) propanoic acid	−6.05 ± 0.02	(36.66 ± 1.26) × 10^3^	−6.22 ± 0.00	(27.43 ± 0.23) × 10^3^	−7.06 ± 0.08	(6.74 ± 0.96) × 10^3^	−5.69 ± 0.10	(68.11 ± 11.52) × 10^3^
DOPAC	−6.51 ± 0.01	(16.79 ± 0.41) × 10^3^	−5.89 ± 0.03	(47.80 ± 2.73) × 10^3^	−7.24 ± 0.02	(4.94 ± 0.12) × 10^3^	−5.43 ± 0.00	(104.84 ± 0.05) × 10^3^
Hippuric acid	−5.14 ± 0.02	(170.32 ± 6.15) × 10^3^	−5.65 ± 0.03	(72.48 ± 3.60) × 10^3^	−6.44 ± 0.01	(19.11 ± 0.21) × 10^3^	−5.48 ± 0.00	(96.57 ± 0.41) × 10^3^
Homovanillic acid	−6.59 ± 0.00	(14.75 ± 0.04) × 10^3^	−6.35 ± 0.00	(22.10 ± 0.12) × 10^3^	−7.14 ± 0.01	(5.79 ± 0.12) × 10^3^	−5.67 ± 0.13	(71.38 ± 15.38) × 10^3^
3-Hydroxyphenylacetic acid	−5.39 ± 0.00	(111.4 ± 1.35) × 10^3^	−5.72 ± 0.00	(64.38 ± 0.49) × 10^3^	−6.29 ± 0.02	(24.67 ± 0.84) × 10^3^	−5.10 ± 0.02	(183.45 ± 5.39) × 10^3^
Vanillic acid	−5.71 ± 0.00	(65.36 ± 0.04) × 10^3^	−5.46 ± 0.01	(100.06 ± 2.15) × 10^3^	−6.01 ± 0.00	(39.29 ± 0.16) × 10^3^	−5.17 ± 0.01	(162.25 ± 3.32) × 10^3^
4-Hydroxyphenylacetic acid	−5.76 ± 0.00	(59.44 ± 0.24) × 10^3^	−5.39 ± 0.04	(111.58 ± 7.46) × 10^3^	−6.73 ± 0.02	(11.56 ± 0.32) × 10^3^	−5.03 ± 0.03	(205.06 ± 10.24) × 10^3^
4-Hydroxybenzoic acid	−4.73 ± 0.00	(339.77 ± 0.19) × 10^3^	−5.05 ± 0.00	(199.56 ± 1.02) × 10^3^	−5.59 ± 0.01	(79.69 ± 1.04) × 10^3^	−4.89 ± 0.01	(261.06 ± 4.91) × 10^3^
3,4-Dihydroxybenzoic acid	−5.45 ± 0.02	(101.37 ± 3.01) × 10^3^	−5.58 ± 0.03	(81.87 ± 4.77) × 10^3^	−6.19 ± 0.00	(29.08 ± 0.01) × 10^3^	−5.30 ± 0.02	(130.06 ± 5.98) × 10^3^
2,4,6-Trihydroxybenzoic acid	−5.28 ± 0.02	(133.88 ± 4.99) × 10^3^	−5.49 ± 0.01	(94.71 ± 1.50) × 10^3^	−6.00 ± 0.03	(42.13 ± 4.46) × 10^3^	−4.96 ± 0.03	(229.49 ± 13.33) × 10^3^
Benzoic acid	−3.72 ± 0.00	(1883.33 ± 9.43) × 10^3^	−4.46 ± 0.00	(537.56 ± 2.44) × 10^3^	−5.34 ± 0.00	(122.32 ± 0.52) × 10^3^	−4.46 ± 0.01	(534.69 ± 8.73) × 10^3^
Phloroglucinol	−4.40 ± 0.00	(598.26 ± 0.29) × 10^3^	−3.88 ± 0.01	(1430.00 ± 21.60) × 10^3^	−4.53 ± 0.00	(482.00 ± 0.08) × 10^3^	−4.67 ± 0.00	(379.82 ± 2.57) × 10^3^
3,4-Dihydroxytoluene	−4.60 ± 0.01	(428.58 ± 6.84) × 10^3^	−4.15 ± 0.02	(904.75 ± 25.33) × 10^3^	−4.60 ± 0.00	(422.91 ± 1.39) × 10^3^	−4.22 ± 0.00	(807.56 ± 1.95) × 10^3^

**Table 2:** Molecular docking of isorhamnetin, quercetin, and MCC950, as the positive control, as well as redocking of ADP to the NLRP3 inflammasome protein. The interactions of the co-crystallized ADP are shown for reference.

Compounds	Binding Energy (kcal/mol)	Hydrogen Bonds and Polar Interactions	Hydrophobic and Aromatic Interactions
Isorhamnetin	−7.09 ± 0.08	Tyr166, Thr167, Ile232, Tyr379, Pro410	Arg 165, Leu233
Quercetin	−8.53 ± 0.03	Phe146, Arg165, Tyr166, Thr167, Gly229	Leu 162, Leu 169, Ile232, Leu233, Arg235, Lys236
MCC950	−11.4 ± 0.00	Glu150, Gly227, Ile228, Gly229, His520	Leu169, Ile232, Leu233, Phe371, Tyr379, Trp414
ADP (redocking)	−8.85 ± 0.04	Arg165, Thr167, Ile228, Gly229, Lys230, Thr231, Ile232, Trp414, His520	Leu169, Phe371, Pro410
ADP (co-crystallized)	-	Thr167, Ile228, Gly229, Lys230, Ile232, Trp414, His520	Phe371, Pro410

## Data Availability

The original contributions presented in this study are included in the article. Further inquiries can be directed to the corresponding author.

## Supplementary Materials

The following supporting information can be downloaded at: https://www.mdpi.com/article/10.3390/ph19050655/s1, Table S1: Chosen proteins with their amino acid residues and coordinates for molecular docking analysis.

## Abbreviation

The following abbreviation is used in this manuscript:

DOPAC	3,4-dihydroxyphenylacetic acid
