# Supplementary material for: In Silico and In Vitro Evaluation of Quercetin Metabolites Binding to Inflammatory Target Proteins
Source: Pharmaceuticals (Basel). 2026 Apr 22;19(5):655. doi: 10.3390/ph19050655 (PMC13210167; doi:10.3390/ph19050655)
Supplement: Supplementary file 1 [file pharmaceuticals-19-00655-s001.zip › pharmaceuticals-4213596-supplementary.pdf]

Table S1. Chosen proteins with their amino acid residues and coordinates for molecular docking analysis.

Protein	Amino acid residues	Grid box coordinates
NLRP3 (pdb: 6npy)	Phe146, Ile149, Leu162, Arg165, Tyr166, Thr167, Leu169, Gly227, Gly229, Thr231, Ile232, Leu233, Arg235, Lys236, Leu233, Lys236, His 258, Arg260, Tyr379, Pro410, Leu411, Trp 414, Asn504, Leu505, Ile519, His520, and Met521	x = 79.595, y = 96.502, and z = 94.028. GP: x = 52, y = 52, and z = 50. The spacing: 0.500 Å.
NF-κβ (pdb: 1a3q)	Lys53, Lys90, Glu92, Asp94, Leu95, Arg103, Ala104, His107, Leu109, Ser115, Glu116, Leu117, Glys118, Ile119, Ala121, Lys153, Arg156, Arg160, Ser161, Arg193, Phe197, Ser206, Pro208, and Ser225	x = 29.358, y = 79.653, and z = -25.026. GP: x = 54, y = 54, z = 54. The spacing: 0.500 Å.
COX-1 (pdb: 6y3c)	His43, Gln44, Arg 61, Arg79, Arg83, His90, Arg120, Asn122, Leu352, Tyr355, Arg374, Asn375, Phe381, Lys468, Arg469, Met472, Lys473, Ile523, Ala527, and Lys532.	x = -22.274, y = -50.999, and z = -0.720. GP: x = 74, y = 76, z = 94. The spacing: 0.414 Å.
COX 2 (pdb: 5f19)	Cys36, His39, Gln42, Asn43, Arg44, Cys47, Thr60, Arg61, Thr62, Thr76, Lys79, Leu80, Lys83, Pro86, His90, Tyr115, Val116, Thr118, Ser119, Arg120, Ser121, His122, Ile124, Asp125, Ser126, Tyr130, Asp133, Gly135, Tyr136, Ala151, Pro156, Asp157, Phe209, Val349, Leu352, Ser353, Tyr355, Gln370, Gln372, Asn375, Phe381, Lys468, Arg469, Met471, Phe518, Phe529, and Gly533	x = 22.923, y = 52.040, and z = 54.981 GP: x = 88, y = 68, and z = 70. The spacing: 0.558 Å.
TNF-α (pdb: 2az5)	Lys11(A/B), His15 (A), Leu57(A/B), Tyr59(A/B), Arg82(B), Tyr87(B), Ile97(B), Tyr119(A/B), Leu120(A/B), Gly121(A/B), Gln125(B), Tyr151(A/B), and Leu157(A/B)	x = -7.583, y = 70.748, and z = 27.399 GP: x = 88, y = 90, z = 10. The spacing: 0.503 Å.
TNFR1 (pdb: 1ext)	Phe60, Ala62, Asn65, His66, Leu67, Leu71, Ser74, Lys75, Arg77, Asp93, Cys96, Glu109, Asn110, and Leu111	x = 1.268, y = 35.608 and z = -6.803 GP: x = 48, y = 48, anz = 48. The spacing: 0.503 Å.
IL-6 (pdb: 1alu)	Glu42, Arg68, Glu79, Glu95, Val96, Glu99, Gln116, Pro141, and Asn144	x = 12.196, y = -23.112, and z = 10.444 GP: x = 46, y = 50, and z = 88. The spacing: 0.492 Å.
IL-6R (pdb: 1n26)	Glu34, Lys45, Pro46, Ala47, Arg54, Arg65, Leu69, Ser72, Asn74, Leu90, Asp92, Ser122, Leu123, and Thr124	x = 41.547, y = 52.121, and z = 47.121 GP: x = 54, y = 74, and z = 88. The spacing: 0.492 Å.
IL-1β (pdb: 5r8q)	Met20, Tyr24, Glu25, Leu26, Gln48, Lys63, Thr79, Leu80, Gln81, Leu82, Met95, Lys97, Val100, Pro131, Val132, Phe133, and Leu134	x = 30.897, y = 13.487, and z = 67.202 GP: x = 52, y = 50, and z = 50. The spacing: 0.497 Å.
IL-1R1 (pdb: 1itb)	Arg9, Glu10, Glu11, Lys12, Ile13, Leu15, Ser18, Arg25, Pro26, Cys27, Pro28, Leu29, Asn30, Asn32, Glu33, Glu62, Lys63, Ile92, Glu98, Asn99, Lys112, Lys114, Tyr127, Met128, Glu129, Phe130, Leu138, Lys161, Asp162, Arg194, Asn204, Gln236, Leu237, Ser238, Ile240, Ile250, Glu252, Tyr261, Ser263, Lys298, and Thr300	x = 34.659, y = 0.661, and z = 17.726 GP: x = 94, y = 58, and z = 62. The spacing: 0.642 Å.

GP: Grid points.
